# A review of co-culture models to study the oral microenvironment and disease

**DOI:** 10.1080/20002297.2020.1773122

**Published:** 2020-06-04

**Authors:** Sophie E Mountcastle, Sophie C Cox, Rachel L Sammons, Sara Jabbari, Richard M Shelton, Sarah A Kuehne

**Affiliations:** a EPSRC Centre for Doctoral Training in Physical Sciences for Health, University of Birmingham, Birmingham, UK; b School of Dentistry, University of Birmingham, Birmingham, UK; c School of Chemical Engineering, University of Birmingham, Birmingham, UK; d School of Mathematics, University of Birmingham, Birmingham, UK; e Institute of Microbiology and Infection, University of Birmingham, Birmingham, UK

**Keywords:** Co-culture, oral disease, oral cavity, biofilms, NC3Rs, 3D-*in vitro* models

## Abstract

Co-cultures allow for the study of cell–cell interactions between different eukaryotic species or with bacteria. Such an approach has enabled researchers to more closely mimic complex tissue structures. This review is focused on co-culture systems modelling the oral cavity, which have been used to evaluate this unique cellular environment and understand disease progression. Over time, these systems have developed significantly from simple 2D eukaryotic cultures and planktonic bacteria to more complex 3D tissue engineered structures and biofilms. Careful selection and design of the co-culture along with critical parameters, such as seeding density and choice of analysis method, have resulted in several advances. This review provides a comparison of existing co-culture systems for the oral environment, with emphasis on progression of 3D models and the opportunity to harness techniques from other fields to improve current methods. While filling a gap in navigating this literature, this review ultimately supports the development of this vital technique in the field of oral biology.

## Introduction

**Figure 1.**
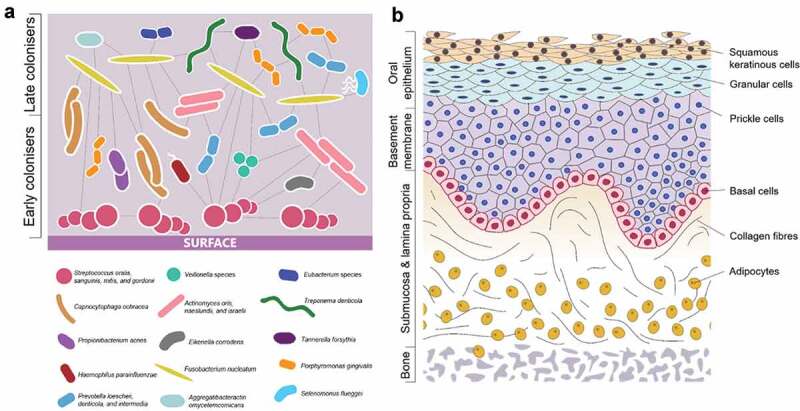
(a) Common bacterial species present in pathogenic oral biofilms and their communication between species (adapted from Parashar et al. [92]). (b) Cells and tissue types present in the oral mucosa, demonstrating complexity of 3D structure.

The oral cavity is a complex environment that contains many microbial species that thrive in the warm, moist conditions [1] (Figure 1a). Furthermore, different regions of the oral cavity are made up of several cell types and tissues, both soft (mucosa, connective tissue, smooth muscle) and hard (enamel, dentine, bone) [2,3] (Figure 1b). Changes in the soft tissues can indicate disease, for example periodontitis (severe gum disease) and oral cancer, and reveal systemic conditions such as diabetes or vitamin deficiency [3]. Equally, the mineralised structures within the mouth may bear signs of disease, including dental caries, that might result in significant hard tissue loss or damage [4]. The composition of microbial species in the mouth can either cause or intensify many of these diseases [5], thus demonstrating the importance of balance within this complex multi-cellular environment.

The microorganisms present in the oral cavity attach to surfaces in communities called biofilms; highly regulated and organised interspecies habitats that provide defence against competitors and adapt to changes in the wider environment [5]. These communities are essential for many metabolic, physiological, and immunological functions. They support food digestion, regulation of the host immune system, maintenance of mucosa barrier function, detoxification of environmental chemicals, and prevent invasion of disease-promoting species [5]. However, a shift in the species present in the oral microbiome can unsettle the local environment, switching from a healthy to disease state [6]. Saliva also plays a key role in the oral cavity in maintaining homeostasis and defending from disease, as well as containing proteins, minerals, and antimicrobial enzymes that control biofilm formation and activity [7,8]. Evidently, understanding the processes and interactions that occur in the oral cavity, in both healthy and disease states as well as the shift between the two, is vital to furthering our knowledge of disease progression and the discovery of new treatments.

For both human and bacterial cells, utilising single species for *in vitro* modelling of the oral cavity does not fully represent the *in vivo* conditions (Figure 1). This presents a key question for researchers in this field regarding how best to study the oral cavity, both for understanding disease pathogenesis and evaluating novel therapeutics. Challenges in studying this complex environment are not just limited to the presence of many cell types and bacterial species, but also the substantial variations in microbiota between individuals [9]. Mimicking these various degrees of complexity remains difficult and therefore *in vivo* studies remain the gold standard for observing processes in oral pathogenesis. However, clinical *in vivo* studies and animal models bring their own obstacles; they are expensive, labour intensive, and can generate ethical concerns. In addition, human and animal oral microbiota may not be the same and therefore can be difficult to compare. As such, the use of co-culture models to mimic *in vivo* conditions has been recognised as a valuable approach to further our understanding of the relationship between eukaryotic and bacterial cells and is especially applicable to the oral cavity.

Co-culture techniques allow a variety of cell types to be cultivated together, enabling examination of cell–cell interactions [10]. These systems may refer to the culture of two or more eukaryotic cell types together, or eukaryotic and prokaryotic cells. The effectiveness of co-cultures is heavily determined by the choice of experimental setup. Cell–cell interactions in co-cultures are strongly influenced by the extracellular environment, which in turn is influenced by the employed protocol [11]. There are numerous factors that need to be optimised to ensure these systems are representative of the native oral cavity, such as the number of cell populations. Having more than two species can result in unstable systems due to multiple reaction pathways, which may be difficult to monitor, analyse, and interpret [11].

Studying the relationship between the oral microbiome and eukaryotic cells is essential to understanding disease progression and evaluating the effect of new treatments. Many studies have published co-culture methodologies, but to the authors’ knowledge, these techniques have not been directly compared, making it challenging to identify and optimise the most appropriate system for a research question. Hence, this review discusses the use of co-culture *in vitro* models to study the oral environment, the progression of these models in complexity, and the disadvantages and benefits of using a range of published methods (Table 1). In addition, the lessons and approaches that can be adapted from other fields that regularly utilise co-cultures are considered with the aim of providing future insights for development. Searches were performed across Science Direct, ProQuest, and the Directory of Open Access Journals [12–14] for papers that reported co-culture studies containing both eukaryotic and bacterial cell species.

**Table 1.** Summary of co-culture methodologies, common protocols employed, and the advantages and disadvantages of each model system.

Method	Summary of protocol	Advantages	Disadvantages	References
2D monospecies co-culture with planktonic bacteria	Seed eukaryotic cells into well plate. Culture until confluent monolayer formed, with media changes every 1–2 days. Prepare bacteria overnight culture. Centrifuge overnight and re-suspend bacteria in eukaryotic cell culture media to achieve desired concentration. Add media containing bacterial suspension to monolayers and perform assays at desired time points.	Can use simple assays to investigate Can attribute direct cellular responses from interactions with bacteria Reproducible with reduced batch-to-batch variation Supports homogenous growth All cells have equal access to nutrients	Not representative of *in vivo* tissue structure Does not account for immune cells Does not account for many cues found *in vivo*, including mechanical signalling Cannot monitor interaction between cell types, in particular, the immune system Does not represent the complex bacterial biofilms present in the oral cavity	[15–17,19,33,34]
2D multispecies co-culture with planktonic bacteria	Seed appropriate ratio of eukaryotic cells into well plate. Culture until confluent, with media changes every 1–2 days. Prepare bacteria overnight culture. Centrifuge overnight and re-suspend bacteria in eukaryotic cell culture media to achieve desired concentration. Add media containing bacterial suspension to cell culture and perform assays at desired time points.	Can monitor the interaction between cell types Reproducible with reduced batch-to-batch variation Supports homogenous growth All cells have equal access to nutrients	May require optimisation due to different nutrient requirements Not representative of *in vivo* tissue structure Traditional assays cannot always determine between cell species Does not account for many cues found *in vivo*, including mechanical signalling Does not represent the complex bacterial biofilms present in the oral cavity	[29,30]
2D co-culture with biofilm	Seed appropriate ratio of eukaryotic cells into well plate. Culture until confluent, with media changes every 1–2 days. Prepare bacteria overnight culture. To form biofilm, seed overnight culture onto coverslips placed in the bottom of a well plate. Change media every 1–2 days. At chosen time point, once biofilm has formed, remove media and attach coverslip to base of transwell insert. Place insert into cell-culture plate. Perform assays at desired time points.	Can monitor the interaction between cell types Reproducible with reduced batch-to-batch variation Supports homogenous growth All cells have equal access to nutrients More clinically relevant, as biofilms show increased antibiotic resistance to planktonic cultures.	Bacteria can overrun eukaryotic cells if co-culture system is not carefully designed	[46,55–57,66,67]
3D tissue engineered co-culture with planktonic bacteria	For collagen-based system, mix fibroblasts with collagen gel and pipette into transwell inserts. Set gel in incubator at 37°C for 1 hr. Seed epithelial cells onto surface of gel. Seed monolayer of epithelial cells into separate well plate to monitor confluence. Culture cells until confluent monolayer formed. Raise cells to air-liquid interface and culture for 7–10 days to allow stratified epithelium to form. Prepare bacteria overnight culture. Centrifuge overnight and re-suspend bacteria in eukaryotic cell culture media to achieve desired concentration. Add media containing bacterial suspension to 3D cell culture and perform assays at desired time points.	More representative of *in vivo* environment Can study cell-cell signalling Two mucosa models well established in literature – collagen-based and decellularised matrix	Can be challenging to achieve cell numbers required for multiple models Require specifically enriched media Significant optimisation may be needed More resource-intensive More difficult to produce replicates Models may not be fully representative of native tissue structure Does not represent the complex bacterial biofilms present in the oral cavity	[41–44,46,47]

## 2D cell culture

**Figure 2.**
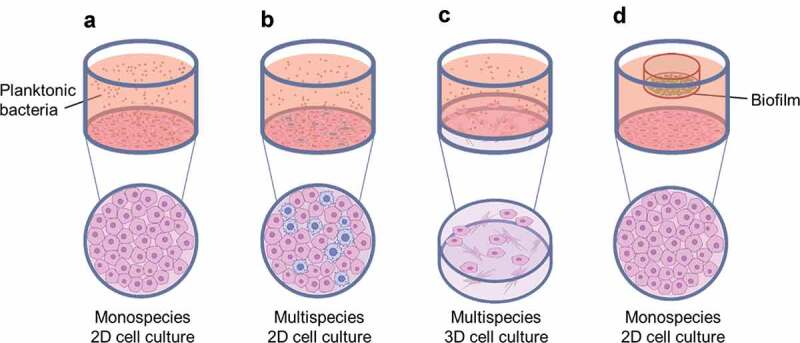
Common co-culture systems reported in the literature (a) monospecies 2D cell culture with planktonic bacteria applied; (b) multispecies 2D cell culture with planktonic bacteria applied; (c) multispecies 3D cell culture, typically a collagen-based or decellularised matrix containing fibroblasts, with planktonic bacteria applied; and (d) monospecies 2D cell culture with biofilm applied, typically suspended from a well insert.

The simplest oral environment co-culture systems apply planktonic bacteria to a monolayer of confluent eukaryotic cells [15–18] (Figure 2a). Compared with more complex approaches, these basic models have an advantage in that cellular response to bacteria can be attributed to specific interactions allowing for direct comparison between species, both bacterial and cellular. For example, the inflammatory response of epithelial cells to different bacterial species may be compared [16] or different eukaryotic cell lines may be challenged with the same oral pathogenic species, such as *Porphyromonas gingivalis* (*P. gingivalis*), a key contributor to the pathogenesis of periodontitis [19]. However, it is known that interactions between different bacteria can affect disease progression [20,21] and therefore applying single species cannot elucidate more complex physiological interactions.

A number of innovative studies utilised 2D co-culture systems to study the adhesion and invasion of epithelial cells by key oral pathogens. *Aggregatibacter actinomycetemcomitans* (*a. actinomycetemcomitans*, formerly *Actinobacillus actinomycetemcomitans*) is a bacterium associated with aggressive periodontitis. Mintz and Fives-Taylor [22,23] applied *a. actinomycetemcomitans* to an oral cancer cell line under different conditions and highlighted that adhesion is affected by both host (saliva, serum) and culture (pH) conditions. Using a similar approach, Yilmaz et al. [24] cultured primary gingival epithelial cells and introduced *P. gingivalis* and its fimbriae-deficient mutant, demonstrating that *P. gingivalis* fimbriae promote adhesion to gingival epithelial cells through interaction with β1 integrins. In a later study, Yilmaz et al. [25] showed that *P. gingivalis* is capable of targeting specific epithelial cell pathways during invasion and can adapt to an intracellular environment. They suggested that disease may ensue from a disruption of the balance between the bacteria and host cells by factors that may trigger virulence or lead to host-immune-mediated tissue damage [25]. Studies like these are essential to determine key proteins and interactions involved in oral pathogenesis, which could potentially provide targets for future treatments.

In addition to looking at a specific bacterium, 2D co-culture systems can be effectively used to compare the response of host cells when challenged with different oral pathogens. Han et al. [26] individually applied six key Gram-negative anaerobic bacteria associated with periodontal diseases to human gingival epithelial cells to compare their ability to adhere and invade, as well as measuring levels of interleukin-8 (a proinflammatory cytokine) secretion from the human cells. Their findings demonstrated that whilst all bacteria species were able to adhere to oral epithelial cells, only *Fusobacterium nucleatum* (*F. nucleatum*) was highly invasive, to levels comparable with *P. gingivalis* [26]. Not only can comparisons be made between different bacteria species using multiple 2D co-cultures, but the ability of different strains to adhere and invade oral epithelial cells can be investigated. The *Prevotella intermedia* (*P. intermedia*) group are made up of three strains (*P. intermedia, Prevotella nigrescens*, and *Prevotella pallens*) and are connected with oral disease pathogenesis. Gursoy et al. [27] showed that *P. intermedia* and *P. nigrescens* type strains can adhere to and invade epithelial cells, the capability of *P. intermedia* being highest. Another key publication in which strains were compared, was the work of Dabija-Wolter et al. [28] who examined the invasion of human gingival fibroblasts by three different *F. nucleatum* strains using a 2D co-culture system. In order to evaluate the amount of bacteria present inside the fibroblasts after infection, live bacteria were fluorescently stained prior to being introduced into the co-culture, and this allowed for visualisation using confocal laser scanning microscopy and quantification using flow cytometry. The studies described use a range of assays and analytical techniques to determine key interactions between host cells and pathogenic bacteria, showing the importance of simple 2D co-culture systems, as well as the influence of strain, cell type, and culture conditions.

To elevate 2D co-cultures and gain further insight into *in vivo* interactions, multiple eukaryotic species can be cultured together (Figure 2B). In two studies by Bodet et al. [29,30], epithelial cells were cultured alongside macrophages to gain a better understanding of the interplay between these two cell types in the presence of *P. gingivalis*. Careful optimisation of the ratio between cell types is essential and consideration should be given to the analytical techniques applied. In these studies, Bodet et al. [29,30] were unable to identify which cells had a greater role in IL-6 and IL-8 secretion. This highlights that more complex assays, such as flow cytometry, may be required to target each cell type. Recently, a three-cell co-culture was described whereby dendritic cells, gingival epithelial keratinocytes, and T-cells were cultured in a three-cell transwell co-culture plate, essentially allowing for three mono-layers to be cultured in the same well and therefore allowing interactions to be determined when challenged with *P. gingivalis* [31]. Different single- and co-cultures were prepared to compare the production of matrix metalloproteases (MMPs) in response to the pathogen. Interestingly, the cellular reaction changed when T-cells were present with a reduction in MMP9 and a reduced immune response, which indicated that multiple cell types could influence MMP expression, thus providing further evidence of the complex cell-cell signalling occurring *in vivo*.

Not only can 2D co-cultures elucidate information on interactions between oral eukaryotic cells, they can also be used to evaluate microbial communication. Several authors have employed 2D co-cultures to study the effect of multiple oral bacterial species on the invasion of gingival epithelial cells by respiratory pathogens [15] and *P. gingivalis* [17]. Findings suggested that commensal oral species could modulate invasion. Providing careful consideration is given to the controls used, a 2D co-culture system with multiple bacterial species can determine very useful information on the pathogenesis of oral disease. From the *in vitro* study described [15], the authors suggest that increased presence of oral bacteria in the throat could prevent invasion of respiratory pathogens. However, it is important to recognise that these co-culture models are not physiologically representative, due to a lack of host immune system and the use of monolayer cell cultures. Therefore, extrapolating the results of such studies to *in vivo* conditions should be done with care.

Interactions of anaerobic species with human cells raise challenges in culturing these bacteria with oxygen-requiring epithelial cells. One of the limitations in the literature described is the culture of *P. gingivalis* in aerobic conditions. Bodet et al. [29,30] and Saito et al. [17] did not report viability of *P. gingivalis* under the growth conditions applied when co-cultured with their respective oral mucosa model. The growth of *P. gingivalis* under oxygenated environments has been shown to affect its physiology and result in changes in expression of different proteins, including virulence factors [32]. Gursoy et al. [27] also highlighted the tolerance of *P. intermedia* strains to oxygen exposure as a limitation of their co-culture study. The test conditions applied were aerobic, and the type strain had been handled in laboratory conditions for longer than the clinical isolates. Consequently, increased tolerance to oxygen exposure of the type strain may have explained their findings of increased adhesion. It is vital to assess and report the effect of the aerobic growth conditions used on anaerobic species for the duration of the experiment.

Simple 2D co-cultures prove useful for testing responses to a dental material, for example, implants or resins. Human gingival fibroblasts can be cultured directly onto the surfaces of these materials, with planktonic oral species added subsequently to investigate their effect. Using this method, oral bacteria have been shown to modulate toxicity of dental resins on human gingival fibroblasts (HGFs) [33]. It is also possible to adapt these 2D co-cultures to enable high throughput studies to be performed in 96-well plates. For example, a study by Giulio et al. [34] reported the effect of dental resin monomers on HGFs in the presence of *Streptococcus mitis* (*S. mitis*) and demonstrated there was no reduction in bacterial adhesion to the eukaryotic cells. Simple 2D cultures also allow for the interaction between cells and dental resin materials (e.g. HEMA) to be studied in the presence of oral microbes, an important interaction to understand in the context of the oral environment [35].

A key factor to consider when using a co-culture system containing eukaryotic cells is their origin. A range of cell types have been used in the studies described, including primary human gingival epithelial cells [16,24–26] and fibroblasts [33,34], immortalised human gingival cell lines [18], oral carcinoma cell lines [15,17,22,23,26], and skin keratinocyte cell lines [27,29,30]. Some studies did not take the source of their human cells into account when discussing their findings. However, oral keratinocytes and fibroblasts show distinct characteristics to those derived from the skin [36,37]. In addition, whilst cell lines are a convenient choice for these *in vitro* systems as they are highly proliferative and easier to culture, they often have phenotypic, morphological, and genetic differences to their primary tissue origin. Primary cells, on the other hand, maintain many of the markers and functions seen *in vivo* and are therefore useful for elucidating responses from human cells when challenged with oral pathogenic bacteria.

The publications described have demonstrated that a simple 2D co-culture model ensures that subsequent assays and analyses are easier to perform and less complex analytical techniques can be used. They also allow for specific interactions to be identified, which is important when investigating disease progression and potentially identifying new therapies for oral pathogenesis. However, there are challenges associated with using simpler models. In particular, neglecting the effects of the host immune system and not representing the 3D structure of *in vivo* tissues mean these models lack certain signals that are present in the body (Table 1).

## 3D cell culture

As we have gained an understanding of the importance of cues from the surrounding environment, such as mechanical and biological signalling between cell types [38–40], there has been a move to mimic the structure of the tissue in which the eukaryotic cells are located (Figure 2C). *Candida albicans* (*C. albicans*) is a commensal yeast that can shift to become pathogenic in immunosuppressed individuals and is therefore an important oral pathogen. A number of 3D *in vitro* culture systems have been developed to mimic the oral mucosa in order to study the interaction between epithelial cells and *C. albicans* [41,42]. The 3D models commonly utilised in these investigations comprise a fibroblast-containing collagen gel with oral keratinocytes cultured on the surface at the air-liquid interface. An alternative to the collagen model is the use of decellularised matrix as a 3D scaffold. Interestingly, Yadev et al. [43] demonstrated that a 3D tissue engineered oral mucosa model of human keratinocytes and a fibroblast-containing matrix displayed more similar immunohistological and proliferation characteristics to normal mucosa when compared with a 2D oral cell line. In this study, full-thickness oral mucosa models were prepared from decellularised human matrix and compared with collagen-based 3D mucosa models purchased from SkinEthic Laboratories (Nice, France) and MatTek Corporation (Ashland, MA).

Surprisingly, there are relatively few 3D oral mucosa co-culture studies that have been applied to model bacteria relevant to oral disease. Of those that have, Pinnock et al. [44] reported significant differences in the response of oral mucosa models to *P. gingivalis*, compared with monolayer cultures of epithelial cells. This study described their use of a collagen-fibroblast gel with surface epithelial cells cultured at the air-liquid interface, with the application of *P. gingivalis* in planktonic culture. Subsequently, it was shown that utilising 3D co-culture systems was important in order to fully discern cellular responses to infection and confirmed that the interaction between cell types played an important role. Another key study that supported the significance of 3D co-cultures in the field of oral pathogenesis investigated the bacterial species *F. nucleatum*, which is known to form a bridge between early and late colonisers in the formation of dental plaque (a common oral biofilm) [45]. Gursoy et al. [46] used a collagen-based 3D mucosa model and applied planktonic cultures of *F. nucleatum* to determine the bacteria’s ability to attach to and invade epithelial cells. Like Pinnock et al. [44], they also highlighted the difference in response between the 3D co-culture and a simple monolayer of epithelial cells. Given the strong evidence of an interplay between epithelial cells and fibroblasts in response to infection, there is a clear need for future studies to consider the application of 3D mucosa models to studies of oral disease pathogenesis [44,46]. Furthermore, it is worth highlighting that both Pinnock et al. [44] and Gursoy et al. [46] reported that the viability of the anaerobic species they utilised (*P. gingivalis* and *F. nucleatum* respectively) was not reduced under aerobic growth conditions for the duration of their infection co-culture model. It is essential to examine the oxygen tolerance for anaerobic species when applying them to oxygen-requiring epithelium models to ensure physiology is not affected. One of the challenges with developing 3D cultures is that primary cells have relatively short lifespans, as they lose their *in vivo* phenotype after a few passages, and therefore may not offer sufficient cell numbers to use in multiple 3D co-cultures [47]. Furthermore, enriched media specific to each cell type are often required; without this, primary cells can display an altered phenotype and metabolic function [48]. To combat these drawbacks, immortalised cell lines of human gingival keratinocytes (HGKs) and human gingival fibroblasts (HGFs) have been established. Promisingly, Bao et al. [47] have demonstrated that immortalised HGKs still formed a stratified epithelial layer and both HGKs and HGFs displayed cell-specific markers similar to those found in human gingival tissues. The need for reproducibility makes the use of cell lines desirable, although it must be noted that there is a pay-off between reproducibility and physiological relevance, with Yadev at al. highlighting that the commercially available epithelial cell line TR146 does not form a fully differentiated epithelium [43].

As with 2D co-culture systems, the origin of the human eukaryotic cells in a 3D mucosa model is an important aspect to consider when analysing the cellular response to bacteria. A range of cell sources were utilised in the co-culture studies described. These included primary cells from gingival biopsies [43,44], immortalised gingival keratinocyte and fibroblast cell lines [46,47], oral carcinoma cell lines [42,43], human skin epithelial cell lines [41,46] and 3T3 cells (mouse embryonic fibroblast cell line) [41]. Not only do these cells exhibit different phenotypes and morphologies but moreover, the choice of fibroblast origin can influence the characteristics of the keratinocytes in a 3D model. Merne & Syrjänen [49] highlighted the importance of standardising the matrix, both in terms of extracellular matrix components and in the source of fibroblasts used. Where possible, human eukaryotic cells should be utilised since they are the most physiologically relevant with regards to the *in vivo* tissue of interest.

An additional factor that needs to be taken into account regarding the application of oral pathogens in 2D and 3D co-cultures is the strain of bacteria utilised. Many of the studies cited throughout this review do not detail the origin of the bacteria used. However, it has been previously shown that there is a difference in keratinocyte response between clinical and type strains of *a. actinomycetemcomitans* [50]. Therefore, it is important to appreciate that strains of the same bacterial species may have varying characteristics. It is advisable, where possible, to use clinical strains as well as type strains to conduct co-culture studies in order to compare them with their standards.

The choice to use a 3D culture needs to be a carefully considered decision, as there is currently no universal system available and therefore significant optimisation may be required [51]. Moreover, 2D cell culture approaches can still provide useful information to enhance our understanding of *in vivo* processes. As well as being easier to reproduce and less resource-intensive, 2D cell cultures support homogenous growth and equal access to nutrients for all cells present, whilst cells embedded in a 3D system may not have access to sufficient nutrients [51]. Despite the challenges that come with 3D systems, the studies cited demonstrate that 3D co-cultures are highly valuable, as monolayer culture systems do not fully represent the high complexity of the oral cavity (Table 1). A review of 3D oral mucosa models by Moharamzadeh et al. [52] described the different approaches that have been taken and the advantages and limitations of each, as well as the range of applications for these systems. As protocols and analysis methods continue to improve, these 3D techniques will become more accessible within the oral field.

## Biofilms

The studies described thus far have utilised bacteria in the form of planktonic cultures, applied within nutrient media to the 2D and 3D cell cultures. Often only one or two bacterial species are considered in these studies, compared to the 700 species that have been detected in the oral cavity [53]. Bacteria in the mouth mostly exist in the form of polymicrobial biofilms, which are particularly relevant when looking at plaque-related pathogenesis [5]. Furthermore, species growing in biofilms have been shown to have higher resistance to antibiotics when compared with planktonic bacteria [54]. This highlights that applying biofilm models in co-culture studies is particularly relevant to mimicking the oral cavity, both for studying disease progression and evaluating antimicrobial approaches. Millhouse et al. [55] showed there is interplay between a complex biofilm and oral epithelial cells, determined through changes in pro-inflammatory mediators. Other studies have similarly revealed pro-inflammatory responses of epithelial cells after challenge with biofilms [56,57]. These investigations demonstrated that specific interactions occur between bacteria in a biofilm, as well as with the host cells, yet these interactions are not present in a planktonic culture. Therefore, it may be concluded that the application of biofilms in co-culture studies with oral eukaryotic cells is essential to unearth the complexity of these microenvironments.

Biofilm models are useful to evaluate anti-inflammatory and antimicrobial techniques, treatments, and compounds. Traditionally, the efficacy of novel antimicrobial compounds is assessed on pathogens in planktonic and biofilm states and subsequently these compounds are applied to oral eukaryotic cells to identify any cytotoxic (or beneficial) effects. This approach is very common in *P. gingivalis* research, as this pathogen is known to induce a response in several oral cell types including epithelial cells, osteoblasts, and fibroblasts [58]. Hence, many studies have an interest in the oral cellular response to novel antimicrobial compounds, as well as the effect on *P. gingivalis* itself [59–61]. However, an area this approach does not address is the interaction between the pathogen and oral eukaryotic cells in the presence of the antimicrobial under investigation.

*P. intermedia* is another potential periodontal pathogen associated with the shift from health to disease in a biofilm and has been shown to increase the immune response at the site of infection [62]. Fteita et al. [18] demonstrated that the chemically synthesised quorum-sensing (QS) molecule butyl-dihydroxy-2, 3-pentanedione, an analogue of autoinducer-2 which is commonly produced by many gram-positive and gram-negative species, was able to reduce cytokine expression of a human gingival keratinocyte cell line and simultaneously inhibit biofilm growth of *P. intermedia*. Without observing the entire system in one *in vitro* study, this synergistic effect may have been missed. To further support the importance of evaluating biofilms and oral cells in co-culture, a study by Ramage et al. [63] applied both single- and multi-species biofilms to an oral epithelial cell line (OKF6/TERT2) and the results implied immune-function changes when varying biofilm composition. They reported the dependence of the immune response on the type of bacterial challenge, further highlighting the complexity of the oral cavity and the need to investigate several different interactions to understand disease pathogenesis and identify novel therapeutic targets.

A challenge with using biofilms in a co-culture is the highly different growth rates between the bacteria and eukaryotic cells [64]. High numbers of bacteria in cell culture can cause rapid nutrient depletion and changes in pH, subsequently hindering the growth of eukaryotic cells [65]. Biofilms contain larger numbers of bacteria compared with planktonic cultures, where the concentration of bacteria can be easily adjusted through dilutions. Hence, it can be useful to adopt a methodology whereby the biofilm does not come directly into contact with the eukaryotic cells, or where flow is present to reduce the bacteria numbers in the co-culture system (Figure 2D). Different approaches have been taken to achieve this. Guggenheim et al. [66] and Thurnheer et al. [67] grew a multispecies biofilm on a hydroxyapatite (HA) disc and placed this upside-down on a ring support that was layered onto a gingival epithelial cell monolayer culture. In contrast, Millhouse et al. [55] and Ramage et al. [63] attached the coverslip on which the biofilm was grown to the base of a transwell culture insert, which was placed within the well plate. Hence, the biofilm was suspended approximately 0.5 cm above the monolayer culture and did not directly come into contact with the oral epithelial cells in the bottom of the well (Table 1). An alternative approach to introducing bacteria is to use a flow chamber. These have been used in some studies to evaluate biofilm formation on implant surfaces [68,69]. To grow biofilms in flow chambers the hydrodynamic conditions must be carefully controlled, ideally akin to saliva flow in the mouth [70]. A review of different biofilm flow methodologies has been described elsewhere [70,71]. Relevant to this review, a recent study utilised a flow chamber to compare adhesion of bacteria versus human gingival fibroblasts on titanium surfaces and determined that the smoothest surface best supported fibroblast adhesion and reduced biofilm formation [69]. These findings highlight that dynamic culture systems remain a promising avenue for further exploration. This is of particular relevance to those studying the oral cavity since it enables the system to mimic saliva flow, thus creating an environment more closely aligned to *in vivo* conditions.

Due to the complexity of analysing both 3D tissue models and biofilms, very few studies have attempted to combine the two in a single system. The most simple reported method, published by Gursoy et al. [46], applied biofilms of *F. nucleatum* grown on coverslips directly onto epithelial cells grown on fibroblast-containing collagen matrices. By comparing the application of planktonic species with biofilms, they were able to determine differences between the ways bacteria behaved in these different states, with biofilm bacteria causing significantly greater epithelial cell death than when applied in planktonic form. This study also demonstrated that cells from biofilms of *F. nucleatum* were able to invade the collagen matrix of the mucosal model, highlighting the benefits of choosing a complex system to model the *in vivo* environment. However, the biofilm was directly in contact with the mucosal model and hence this may have increased the magnitude of the effects observed. A more complex approach to modelling the interaction between oral biofilms and oral tissues is to utilise a perfusion bioreactor system [72,73]. Bao et al. were the first to use one of these systems to study periodontal infections and later also used it to characterise the global proteome regulations present in the host-biofilm model. One of the benefits of using a perfusion bioreactor is that immune cells such as monocytes can be incorporated to generate an environment that is potentially more physiologically relevant. However, the cost of a bioreactor system can be a significant barrier to using this technique. Overall, the research described provides clear evidence that understanding the interactions occurring within an oral biofilm will enhance our understanding of the pathogenesis of oral disease, and novel approaches to introducing biofilms to host cells are key to achieving this.

## Perspectives from other fields

### Gut microbiology

The human intestines exhibit a multifaceted microbiota, with an abundance of host-microbe, microbe-microbe, and environmental interactions [74]. This complexity creates many similar challenges to researchers working in the oral field. Links between the gut microbiome and obesity, diabetes, liver disease, cancer, and neurodegenerative diseases [75,76] have now been established, which has driven considerable growth in this research area. Similar approaches to the co-culture models described herein have been utilised in this field, including monolayer/planktonic cultures [77–80] and 3D/planktonic cultures [81,82]. Reported methods to generate an *in vitro* environment that better represents the *in vivo* surroundings of the digestive tract include bioreactors [79], 3D organoid cultures [83,84], and organ-on-a-chip systems [85], all of which are described in detail by a review published in 2017 [78]. As with certain anaerobic bacteria in the oral field, interactions of anaerobic gut species with the intestinal mucosa are less-frequently studied due to challenges in culturing anaerobes with the oxygen-requiring epithelium. Anonye et al. [86] reported for the first time the use of a dual environment vertical diffusion chamber (VDC) to study the effect of *Clostridioides difficile* (*C. difficile*) on a 3D gut epithelium model. The use of a VDC allowed for monitoring this interaction over a longer time frame and the study reported that *C. difficile* adhered more effectively to epithelial cells grown on the surface of the 3D model than on single epithelial monolayers. VDC could similarly be employed in the field of oral microbiology to better study the effect of anaerobic pathogens such as *P. gingivalis* and *F. nucleatum*, key species identified in the progression of periodontitis.

### Skin microflora

The skin is inhabited by a multitude of microorganisms, with many factors including genetics, environmental characteristics, and host demographics having an influence on the composition of the microflora and consequently on the shift from health to disease [87]. There are a number of 3D skin models established, including some that are available to purchase, which have a fully differentiated epithelium [88]. These models have typically been used in toxicity studies and drug testing applications [89]; however, due to the similarities in structure between skin and oral mucosa, lessons can be taken from some of the advanced approaches to 3D dermal models. For example, El Ghalbzouri et al. [90] demonstrated that collagen secretion by human fibroblasts provided a long-term functional human dermal matrix, and that this could be cultured for nearly three times as long as traditionally used rat-tail collagen matrices. This methodology could be beneficial in the oral field, as the short timespan that primary gingival fibroblasts can be cultured is a limiting factor for longer-term studies of periodontal disease. A similar technique frequently applied in the dermal field is to seed keratinocytes onto decellularised matrix. Anderson et al. [91] were interested in the formation of a biofilm phenotype of MRSA, to mimic a natural infection. They used decellularised porcine vaginal mucosa to generate a stratified, squamous epithelium, an advantage of which is that it is inexpensive and easily reproducible. The same study compared planktonic application of *Staphylococcus aureus* (*S. aureus*) with the formation of a biofilm directly on the skin model and demonstrated the importance of closely mimicking natural biofilm infections. In summary, 3D skin models are becoming increasingly useful in the study of the human dermal microbiota. Some of the novel advances made in this area, in particular the production of 3D extracellular matrix from human fibroblasts, could be translated to the oral mucosa to improve the reproducibility and accessibility of current techniques.

## Conclusions

Significant progress has been made towards the development of physiologically relevant models of the oral environment, from simple 2D co-cultures to more complex 3D tissue constructs and from the application of planktonic bacteria to multispecies biofilms. These advances have led to a greater increase in our understanding of the interactions taking place in the oral cavity, and thus deepening our knowledge of how periodontal diseases progress. However, current *in vitro* models have limitations, either due to their simplicity or complexity. Whilst able to identify specific interactions between cell types, simple 2D cultures cannot be used to determine the more complex cell–cell interactions that occur in the oral cavity, for example, between bacterial species and with the host immune system. On the other hand, due to the analytical complexity or equipment costs, very few studies have successfully introduced biofilms to a 3D organotypic mucosa model. Selecting a co-culture system with an appropriate degree of physiological relevance to answer the research question is essential. As a growing number of studies utilise more complex models, many analytical techniques and 3D mucosa models are being optimised. Utilising knowledge from multiple disciplines, including biology, engineering, and mathematics, is likely to become important in furthering the field due to the multifaceted nature of co-culture systems. Additionally, *in silico* models of interactions in the oral cavity may become of increasing significance for simulating more complex environments, though *in vitro* and *in vivo* data will still be required to make computational approaches reliable. Adapting and applying techniques from other fields facing similar challenges can enhance the methodologies currently available in the study of the oral cavity. Systems that combine these approaches will ensure advancement in the field. As such, this will enhance our understanding of disease progression and enable the evaluation of the effects of new antimicrobial compounds and novel therapies.
